# Pharmacomicrobiomics: a novel route towards personalized medicine?

**DOI:** 10.1007/s13238-018-0547-2

**Published:** 2018-04-28

**Authors:** Marwah Doestzada, Arnau Vich Vila, Alexandra Zhernakova, Debby P. Y. Koonen, Rinse K. Weersma, Daan J. Touw, Folkert Kuipers, Cisca Wijmenga, Jingyuan Fu

**Affiliations:** 1 Departments of Genetics, University Medical Center Groningen, PO Box 30001, 9700 RB Groningen, The Netherlands; 2 Departments of Paediatrics, University Medical Center Groningen, PO Box 30001, 9700 RB Groningen, The Netherlands; 3 Departments of Gastroenterology & Hepatology, University Medical Center Groningen, PO Box 30001, 9700 RB Groningen, The Netherlands; 4 Departments of Clinical Pharmacy & Pharmacology, University Medical Center Groningen, PO Box 30001, 9700 RB Groningen, The Netherlands; 5 Departments of Laboratory Medicine, University Medical Center Groningen, PO Box 30001, 9700 RB Groningen, The Netherlands; 6 K.G. Jebsen Coeliac Disease Research Centre, Department of Immunology, University of Oslo, P.O. Box 1072, Blindern, 0316 Oslo, Norway

**Keywords:** gut microbiome, drug metabolism, personalized medicine

## Abstract

Inter-individual heterogeneity in drug response is a serious problem that affects the patient’s wellbeing and poses enormous clinical and financial burdens on a societal level. Pharmacogenomics has been at the forefront of research into the impact of individual genetic background on drug response variability or drug toxicity, and recently the gut microbiome, which has also been called the second genome, has been recognized as an important player in this respect. Moreover, the microbiome is a very attractive target for improving drug efficacy and safety due to the opportunities to manipulate its composition. Pharmacomicrobiomics is an emerging field that investigates the interplay of microbiome variation and drugs response and disposition (absorption, distribution, metabolism and excretion). In this review, we provide a historical overview and examine current state-of-the-art knowledge on the complex interactions between gut microbiome, host and drugs. We argue that combining pharmacogenomics and pharmacomicrobiomics will provide an important foundation for making major advances in personalized medicine.

## **INTRODUCTION**

Individual responses to a specific drug vary greatly in terms of both efficacy and toxicity. It has been reported that response rates to common drugs for the treatment of a wide variety of diseases fall typically in the range of 50%–75%, indicating that up to half of patients are seeing no benefit (Spear et al., 2001). Moreover, many individuals suffer from adverse drug reactions (ADRs). Approximately 3.5% of hospital admissions in Europe are related to ADRs, and about 10% of all patients in hospital experience an ADR during hospitalization (Bouvy et al., 2015). In the USA, serious drug toxicities cause over 100,000 deaths and cost 30–100 billion USD annually (Sultana et al., 2013). Inter-individual variability in drug response thus affects not only patient well-being, it also poses an enormous clinical and financial burdens. For the development of “personalized medicine”, it is therefore crucial to determine how we can assess a patient’s probable response to a drug, increase drug efficacy and reduce the risk of ADRs.

Over the past several decades, pharmacogenetics and pharmacogenomics have been at the forefront of research examining the impact of individual genetic make-up on drug response variability. It has now been estimated that genetic factors could explain 20%–95% of the variability in response to individual drugs (Kalow et al., 1998). Thus genetic factors alone are insufficient to explain the observed variability and other factors must be involved.

In recent years, the gut microbiota has emerged as an “organ” that plays an important role in health and disease. The human gut harbours thousands of different bacterial species and other microorganisms that form a complex ecosystem. The composition of the gut microbiome shows high inter-individual variation (Huttenhower and Human Microbiome Project Consortium, 2012) that is associated to a number of host and external factors (Falcony et al., 2016; Zhernakova et al., 2016). Various studies in mice and humans have shown the effect of drug intake on the gut microbiome (Forslund et al., 2015; Wu et al., 2017). In turn, the gut microbiome can also contribute to an individual’s response to a specific drug (Routy et al., 2018; Gopalakrishnan et al., 2017): the microbial community in the gut can modify the pharmacodynamics of a medication by directly transforming the drug or by altering the host’s metabolism or immune system.

Understanding the role of the gut microbiome in drug response may enable the development of microbiome-targeting approaches that enhance drug efficacy. The term pharmacomicrobiomics has been proposed to describe the influence of microbiome compositional and functional variations on drug action, fate and toxicity (Saad et al., 2012). Clearly, the gut microbiome is emerging as an essential component in the development of personalized medicine and modulating the gut microbiome has the potential to become a very attractive approach to managing drug efficiency and safety on the level of the individual.

## **THE COMPLEX SYSTEM OF DRUG METABOLISM**

**Figure 1:**
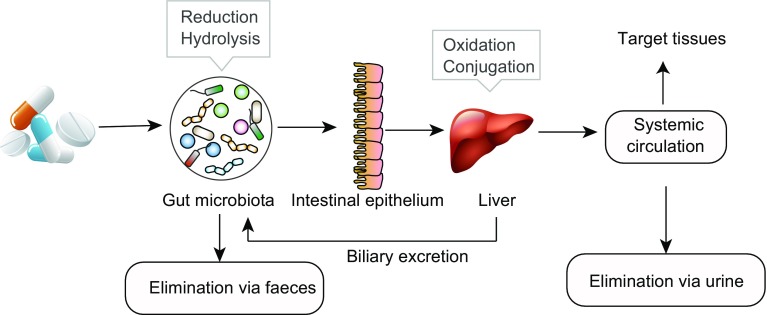
**Sites and types of reactions for drug metabolism**. Bacterial enzymes can participate in drug metabolism mainly through reductive and hydrolytic reactions. Drugs and their metabolites can be absorbed from the intestine and transported via the portal vein to the liver, where a fraction of them will be taken up and another fraction will spill over to the systemic circulation. Hepatic enzymes mainly conduct oxidative and conjugative reactions. Subsequently, drugs and/or their metabolites can be excreted back into the blood to be transported to targeted tissues, removed by the kidney via the urine, or directly excreted by the liver via the biliary system back into the gut lumen

Orally ingested drugs may pass through the upper GI tract and small intestine into the large intestine, where they encounter the thousands of microbial species that reside in the human gut. Complex drug-microbial interactions occur mainly in the colon. Drugs may change intestinal microenvironment, alter microbial metabolism or affect bacterial growth, thereby altering microbial community composition and function. Conversely, the gut microbiome can also participate directly in chemical transformation of drugs (Fig. 1). In the host, drug metabolism occurs predominantly in the liver and can be divided into two phases of reactions: modification and conjugation. It has been noted, however, that the chemical modifications carried out by gut bacteria is very different from these hepatic processes. Gut microbes primarily conduct hydrolytic and reductive reactions to metabolize xenobiotics, while enzymes in the liver typically conduct oxidative and conjugative reactions (Koppel et al., 2017). Upon metabolism in the gut and/or the liver, drug metabolites are either transported to targeted tissues or excreted by the kidneys into the urine or by the liver via the biliary system back into the gut lumen. In the gut, drugs or their drug metabolites can be subjected again to bacterial metabolism (e.g., deconjugation) and (re)absorption (Stein et al., 2010). This complexity means that pharmacological studies require a systems biology approach that considers drug-related hepatic and bacterial metabolic processes, as well as complex host-microbe-drug interactions.

## **MEDICATION PERTURBS THE GUT MICROBIOTA**

Alteration in microbial composition and function by a drug can contribute to the overall effects of that drug on the host, which raises concerns in drug administration. Effects of antibiotics on the gut microbiome are the most studied, and antibiotic-induced dysbiosis in the gut microbiome can increase susceptibility to infections, compromise immune homeostasis, deregulate metabolism and obesity (Francino, 2016). Moreover, it is also a leading cause of *Clostridium difficile* infection, a severe intestinal inflammation caused by the overgrowing of this bacteria, which affects around 124,000 people per year and causes 3,700 deaths annually in Europe (European Centre for Disease Prevention and Control, 2015). Beyond antibiotics, a number of studies in humans and mice have now reported the impact of other commonly used drugs on the gut microbiome. This includes our metagenomics study in a Dutch population cohort of 1,135 samples, where we identified 19 drugs that affected gut microbiota composition (Zhernakova et al., 2016). A similar study in a Flemish cohort (FGFP cohort) reported that nearly 10% of inter-individual variation in the gut microbiome can be explained by medication use (Falcony et al., 2016). The medications identified in both studies were drugs prescribed for treatment of common diseases including gastro-oesophageal reflux, type II diabetes, depression, cardiovascular diseases and hyperlipidaemia.

**Figure 2:**
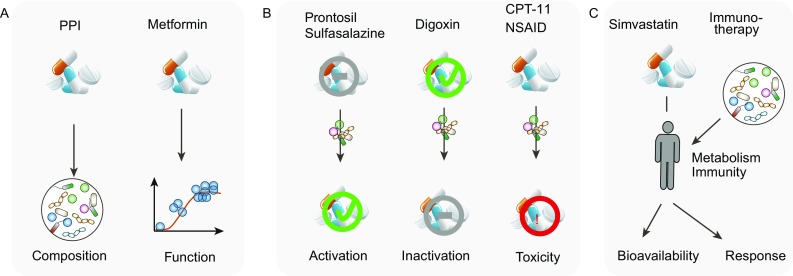
**Drug-microbe effects**. (A) Impact of drugs on the gut microbiome: drugs can perturb microbial composition and function. (B) Direct effect of gut microbiome on drug efficacy and toxicity: microbial transformation can activate or inactivate drugs, or induce drug toxicity to the host. (C) Indirect effect of gut microbiome on drug response: the gut microbiome can influence drug bioavailability and drug response via its interaction with host immune and metabolic systems. Specific examples illustrate each case

While the majority of the current findings are association-based, the identification of a causal impact of proton pump inhibitors (PPIs), which are used to treat gastro-oesophageal reflux and heartburn, and the anti-diabetic drug metformin on gut microbiome composition provides firm evidence that alteration in gut microbiome should be considered when evaluating drug safety and that drug use can also confound microbiome analysis (Fig. 2A).

### **Proton pump inhibitors**

PPIs are commonly used to treat acid-related diseases like gastro-oesophageal reflux disease. Acting through pH-dependent or pH-independent mechanisms, PPIs have the potential to alter the microbiota throughout different parts of the human gastrointestinal lumen (Freedberg et al., 2014). The impact of PPIs on the microbiome is widely reported (Imhann et al., 2016; Jackson et al., 2016). As PPIs reduce acidity in the stomach, there have been reports of overrepresentation of oral microbes in the gut (Imhann et al., 2016), likely due to a reduced stomach barrier function. This reduction in barrier function means pathogenic bacteria may also colonize the gut, and PPI users have a higher risk of enteric infections caused by *Clostridium difficile* (Dial et al., 2004). Interestingly, taxa alterations similar to those associated with *C*. *difficile* infection have also been seen in PPI users, including increased *Streptococcus*, *Enterococcus* and decreased *Clostridiales* (Freedberg et al., 2015). Another study showed that PPIs can accelerate endothelial senescence (Yepuri et al., 2016), although the role of gut microbiome in this adverse event remains unclear. Identification of the strong and unfavourable effect of PPIs on microbiome composition has led to discussions about banning their over-the-counter availability.

### **Metformin**

Metformin is commonly used in the treatment of type II diabetes, and a beneficial impact of metformin in regulating the structure and function of the microbiota is emerging. Forslund et al. were the first to report that metformin could increase the abundance of bacteria that produce short chain fatty acids (SCFA), and these could mediate the therapeutic effects of metformin (Forslund et al., 2015). This observation was also confirmed by the observation of increased faecal levels of SCFAs in metformin users (Zhernakova et al., 2016). Metformin treatment has also been observed to increase the abundances of butyrate-producing bacteria and the mucin-degrading bacteria *Akkermansia muciniphila* (Forslund et al., 2015; Wu et al., 2017; Shin et al., 2014). Transferring human faecal samples from metformin-treated donors to germ-free mice improved glucose tolerance in the mice that received metformin-altered microbiota (Wu et al., 2017).

## **DIRECT IMPACTS OF THE GUT MICROBIOTA ON DRUG EFFICACY AND TOXICITY**

Direct microbial effects on drug response are the chemical transformations of drug compounds by gut microbiota that influence a drug’s bioavailability or bioactivity and its toxicity (Koppel et al., 2017; Spanogiannopoulos et al., 2016) (Fig. 2B). To date, more than 30 drugs have been identified as substrates for intestinal bacteria (Jourova et al., 2016).

Recent insights into biotransformation of drugs by the gut microbiome and the clinical consequences hereof have led to a paradigm shift in pharmacokinetic analyses in humans.

### **Microbiome effects on drug activity**

The first report of a microbial impact on drug activity can be dated back to 1930s with the discovery of the liberation of sulphanilamide via microbial transformation of prontosil (Fuller, 1937). Prontosil is an anti-bacterial drug and was one of a series of azo dyes examined by Gerhard Domagk for possible effects on haemolytic streptococcal infection, work for which he subsequently received the 1939 Nobel Prize in Medicine (Raju, 1999). It was subsequently observed that prontosil had no antibacterial action *in vitro*, and this led to the follow-up discovery in 1937 that its activity is due to the cleavage of the azo bond by bacterial azoreductases and the liberation of sulphanilamide that exerts anti-bacterial activity (Fuller, 1937). Sequentially, several prodrugs were developed with azo bonds that require bioactivation by gut microbes, including sulfasalazine, a drug in the treatment of ulcerative colitis. Bacterial cleavage of azo bonds in sulfasalazine in the intestine can favourably achieve site-specific release of the anti-inflammatory sulfapyridine and 5-aminosalicyclic acid (Peppercorn and Goldman, 1972).

Biotransformation by the gut microbiome can also inactivate drugs, as is seen with the drug digoxin. Digoxin is a commonly used cardiovascular drug, however, in around 10% of patients, the drug is converted to digoxin reduction products that are cardio-inactive. The inactivation of digoxin by gut microbiota was first reported in the 1980s, and antibiotic treatment resulted in a marked increase in serum concentrations of digoxin (Lindenbaum et al., 1981). The underlying mechanism remained unclear, however, until the discovery of specific *Eggerthella lenta* strains in 2013 (Haiser et al., 2013). By combining transcriptional profiling, comparative genomics and culture-based arrays, these *E*. *lenta* strains were identified as carrying a two-gene cardiac glycoside reductase (*cgr*) operon that is transcriptionally activated by digoxin. Arginine is proposed to serve as the main source of nitrogen and carbon for the growth of *E*. *lenta* (Sperry and Wilkins, 1976), while arginine could also inhibit digoxin inactivation (Saha et al., 1983). In line with this, the transcriptional activation of the *cgr* operon has been found to be dependent on arginine concentration. This observation has led to patients being encouraged to eat a high-protein-diet (high-arginine) to block inactivation of digoxin. More recently, Kumar et al. found that the binding pocket of digoxin at the *cgr* operon primarily involves negatively charged polar amino acids and a few non-polar hydrophobic residues and fumarate, which can bind to the same binding sites but with a higher binding energy than digoxin. This knowledge may lead to development of drugs that block *cgr* binding sites (Kumar et al., 2017).

### **Microbiome effects on drug toxicity**

Toxicity occurs when the bacterial transformation of a drug leads to the generation of metabolites that have harmful effects on the host. The role of the gut microbiome on chemotherapy efficacy and toxicity has been recently well discussed (Alexander et al., 2017). One of the best known examples involves the bacterial enzyme β-glucuronidases, which has been described to be involved in the toxicity of the common colon cancer chemotherapeutic CPT-11 (also known as irinotecan). Up to 80% patients using CPT-11 can present with severe diarrhoea. CPT-11 is primarily metabolized in the liver, where human carboxylesterases first activate CPT-11 to its cytotoxic metabolite SN-38, which then inhibits the nuclear topoisomerase 1 enzyme critical for DNA replication. In drug elimination, SN-38 is glucuronidated to its inactive form SN-38G by the liver UDP-glucuronosyltransferase (UGT). SN-38G is excreted via the biliary track into the gut, where bacterial β-glucuronidases can re-activate the drug by converting SN-38G back to SN-38 (Stein et al., 2010), which exhibits toxicity toward intestinal epithelial cells and causes diarrhoea. Via the same mechanism, bacterial β-glucuronidases can also induce toxicity of non-steroidal anti-inflammatory drugs (NSAIDs), which can cause gastroduodenal mucosal lesions in up to 50% of users (Higuchi et al., 2009). When glucuronidated NSAIDs secreted via the hepatobiliary pathway reach the distal small intestinal lumen, bacterial β-glucuronidases produce aglycones that can be taken up by enterocytes. Intestinal cytochrome P450s further metabolize aglycones to potentially reactive intermediates that induce severe endoplasmic reticulum stress or mitochondrial stress leading to cell death (Boelsterli et al., 2013). This mechanism explains the toxic effect of NSAIDs on the intestinal wall.

Because β-glucuronidases are present in a wide range of dominant gut bacteria (Dabek et al., 2008), it is a challenge to design bacterium-specific targets to reduce drug toxicity. Yet modulating activity of bacterial enzymes has become an attractive approach to alleviate drug toxicity. Wallace et al. have identified several β-glucuronidase inhibitors that can efficiently inhibit enzyme activities in living aerobic and anaerobic bacteria while not affecting bacterial growth or harming host epithelial cells (Wallace et al., 2010). Mouse experiments have now shown that oral administration of inhibitors efficiently alleviates drug toxicity of CPT-11, pointing to a potential drug to reduce β-glucuronidase-driven drug toxicity (Wallace et al., 2010).

## **INDIRECT IMPACT OF THE GUT MICROBIOTA ON DRUG RESPONSE**

Indirect microbial effects are microbial influences on drug bioavailability and response via an impact of their metabolic or peptide products on the host immune system or host metabolism (Wu et al., 2017; Gopalakrishnan et al., 2017; Routy et al., 2018) (Fig. 2C). One representative example of microbial impact on drug bioavailability is seen for simvastatin, a drug commonly prescribed in the treatment of hyperlipidaemia. Plasma concentrations of simvastatin are positively associated with microbially synthesized secondary bile acids (Kaddurah-Daouk et al., 2011). Since bile acids are important agents for intestinal nutrient absorption, this also may determine the absorption of simvastatin into the host and influence the drug’s bioavailability.

**Figure 3:**
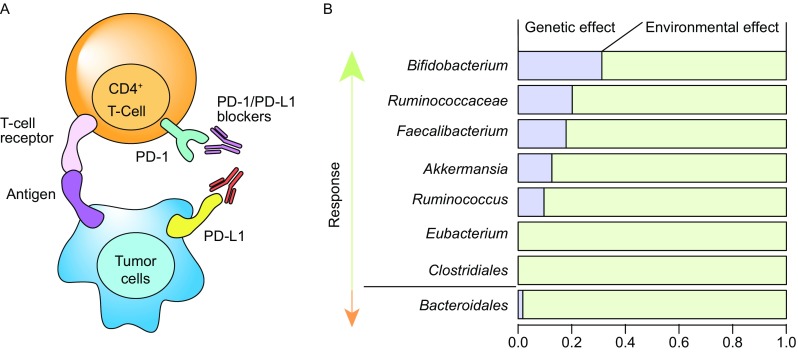
**Gut microbiome associated with response of PD-1/PD-L1 based immunotherapy**. (A) Checkpoints of immunotherapy. Programmed cell death protein 1 (PD-1) is a cell surface receptor that serves as an immune checkpoint. This receptor plays an important role in suppressing T cell inflammatory activity and down-regulating the immune system. Tumour cells can express PD-1 ligands (PD-L1) that are able to bind to PD-l protein and thus inactivate T cells. Accordingly, several PD-1/PD-L1 blockers have been designed to block the interaction between PD-1 and PD-L1 to enable anti-tumour immunity. (B) Gut microbes associated with individual response of immunotherapy and the proportion of their variation explained by host genetics and environmental factors. Five bacterial taxa are associated to higher response, while *bacteroidales* is linked to low response. Inter-individual variation is scaled as 1 and the proportion of explained variation by genetic factors and environmental factors are shaded blue and green, respectively. Estimation of explained variation derived from the TwinsUK study: Goodrich et al. (2014) Cell, 159:789–799

A very intriguing example of microbial impact on drug response can be seen in recent advances in immunotherapy efficacy (Gopalakrishnan et al., 2017; Routy et al., 2018). In oncology, one of the most promising anti-cancer therapies is immunotherapy aimed at alleviation of the blockade of immune checkpoints, using treatments including PD-1/PD-L1 blockers or anti-CTLA4 therapy (Fig. 3A). However, response to these therapies is often heterogeneous. The influence of the gut microbiome on immunotherapy response was first reported in mice by Sivan et al. (2015), who found that commensal *Bifidobacterium* showed a positive association with antitumor T cell response and *Bifidobacterium*-treated mice showed a significant improvement in tumour control. This mouse study led to a follow-up study in human cancer patients (Gopalakrishnan et al., 2017) comparing gut microbial composition in 112 melanoma patients undergoing anti-PD-1 therapy. It showed that patients responding to immunotherapy had higher gut microbial diversity and a higher abundance of *Clostridiales, Ruminococcaceae* and *Faecalibacterium*. This microbiome structure may enhance systemic and anti-tumour immune response via increased antigen presentation and improved effector T cell function. In contrast, the non-responders had lower microbial diversity and a higher abundance of *Bacteroidales*. Another independent study in patients with epithelial tumours also showed that individual response to PD-l/PD-L1 blockers is determined by gut microbiome composition (Routy et al., 2018). In drug responders, over-representation was observed for *Akkermansia*, *Ruminococcus* spp., *Alistipes* spp. and *Eubacterium* spp., while under-representation was found for *Bifidobacterium adolescentis*, *B. longum* and *Parabacteroids distasonis*. This study further explored potential microbiome-modulating therapeutic approaches to enhance drug response and found that simply avoiding antibiotics while taking PD-1 blockers could boost the patient’s positive response from the current 25% up to 40%. Probiotics, like orally administrated *A*. *muciniphila,* can also enhance the response to PD-1 blockers in humans and mice. While further study is still needed to elucidate the underlying mechanisms, it is plausible that this beneficial effect is exerted via the anabolic functions of the gut microbiome, which may promote host immunity. For instance, SCFAs, a major type of bacterial metabolites from dietary fibres, could influence differentiation of T-helper 17 (Th17) and T-regulatory (Treg) cells (Omenetti and Pizarro, 2015). SCFAs have also been implicated in anti-inflammatory properties of *Clostridia* strains (Atarashi et al., 2013; Smith et al., 2013), which would have a beneficial effect on immune function and epithelial permeability (Stefka et al., 2014). Moreover, the gut microbiome has been found to determine an individual’s inflammatory cytokine production in response to different pathogens (Schirmer et al., 2016).

## **COMPLEX GENETICS-DIET INTERACTION IN PHARMACOMICROBIOMICS**

**Figure 4:**
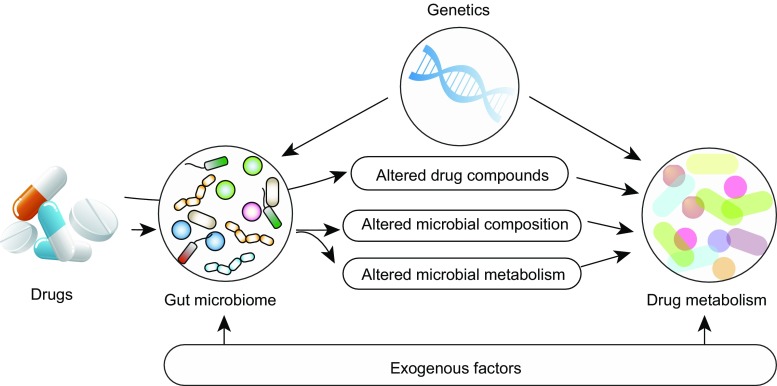
**Host-microbe-diet interactions in drug metabolism**. Complex drug-microbe interactions can result in alterations in microbial composition and function and change the chemical structure of compounds that could directly or indirectly affect drug metabolism in the liver. Moreover, genetics and exogenous factors, including diet, can affect both gut microbiome and drug metabolism in the host

Pharmacomicrobiomic studies that aim to investigate the bidirectional effects between the gut microbiome and drugs need to consider that the gut microbiome is itself a complex trait. It can be affected by host genetics, exogenous factors and by their interactions. Genome-wide association studies (GWAS) have shown that the microbial composition in an individual’s gut can be affected by genetic variants involved in innate immunity, metabolism and food processing. Exogenous factors like diet also have marked effects on the gut ecosystem: what we eat also feeds our gut microbes. A western diet and lifestyle (i.e., a high calorie, high fat diet and a sedentary lifestyle) is widely reported to be associated with a less diverse microbial ecology than, for instance, a high fibre diet. In addition, we have reported associations of 68 dietary factors to the gut microbiome (Zhernakova et al., 2016). However, our diet also contains bioactive compounds that can interact with drugs. Therefore, there is a tripartite interaction between genetics, the gut microbiome and exogenous factors (including diet) in drug metabolism (Fig. 4).

### **Impact of host genetics on gut microbiome and drug metabolism**

Once a drug is administered, it interacts with targets (such as transporters, receptors and enzymes), may undergo metabolism, and is then removed from the system. Each of these processes could potentially involve clinically significant genetic variants (Touw, 1997). Over the past decades, pharmacogenetic studies have identified numerous genetic variants via GWAS (Spear et al., 2001). For instance, the toxicity of CPT-11 discussed above is not only linked to the gut microbiome (Stein et al., 2010) but also to genetic variants in the *UGT1A1* gene. Around ~10% of the western population carries a genetic variant in *UGT1A1* that leads to poor drug metabolism and results in a higher risk for severe toxicity (Innocenti et al., 2004). Clinical decision-making has been increasingly incorporating information of human genetic variation. It has also been shown that individual tailored drug administration based on genotype of *CYP2C9* and *VKORC1* genes can reduce risk of hospitalization caused by the commonly used anticoagulant warfarin by as much as 30% (Madian et al., 2012).

The impact of host genetics on the gut microbiota has also been emerging. Benson et al. conducted the first QTL-based association study in mouse intercross lines and provided clear evidence for the impact of genetic variation on the gut microbiome (Benson et al., 2010). The heritability of individual bacteria in humans was first estimated in 416 pairs of twins from the TwinsUK cohort (Goodrich et al., 2014). The host genetic influence on the gut ecosystem might also have an impact on microbes involved in drug toxicity and efficacy. If we focus, for instance, on taxa associated with the modulation of PD-1/PD-L1 blocker response in immunotherapy, we see that a large proportion are heritable, for example, *Bifidobacterium* (h^2^ = 0.32), *Ruminococcaceae* (h^2^ = 0.20), *Faecalibacterium* (h^2^ = 0.18), *A*. *muciniphila* (h^2^ = 0.12) (Fig. 3B).

Several GWAS have been conducted in humans to identify individual genetic variants associated to gut microbiome in humans (Goodrich et al., 2014; Bonder et al., 2016; Goodrich et al., 2016; Wang et al., 2016). Despite limited direct overlap of associated loci across different studies, a difference potentially due to different analysis methods and low statistical power, the associated loci from all studies generally converge into several physiological processes involved in innate immunity, metabolism and food processing. In particular, C-type lectin molecules and functional variants in the lactase gene (LCT) have been consistently associated to gut microbiome composition and pathways in several studies (Kurilshikov et al., 2017). This not only highlights the complex host-microbe immune and metabolic interactions, it also provides an opportunity to study the causal role of the gut microbiome in health and disease by using genetic variants as instrumental variables in causal inference analysis (a Mendelian randomization approach) (Sheehan et al., 2008).

### **Impact of diet on gut microbiome and drug metabolism**

Dietary components are extrinsic factors that can affect various physiological processes in humans and gut microbial composition. Many microbial enzymes involved in drug metabolism can also metabolize dietary components. Drug-diet interaction occurs when the consumption of a particular food affects the absorption of a drug or modulates the activity of drug-metabolizing enzymes, resulting in altered pharmacokinetics of the drug. The impact of dietary protein and fat on drug metabolism was first noted in the 1970s (Campbell and Hayes, 1976). With the progress of pharmacogenomics, more insights have been obtained at the molecular level. For instance, some drug-metabolizing enzymes have been found to be very sensitive to dietary effects, including several members of Cytochrome P450 family (CYP3A4, CYP1A2 and CYP2E1) and P-glycoprotein (P-gp) transporters localized at the gut epithelium (Harris et al., 2003; Markowitz et al., 2003; Peters et al., 2016). It has been suggested that dietary factors can alter expression levels of these enzymes/transporters in the intestine, as well as their substrate-specificity, thus affecting drug metabolism by these enzymes.

Diet is one of the most important exogenous factors shaping the gut microbiome. Long-term and short-term effects of dietary factors on the gut microbiome are well-documented (Falcony et al., 2016; Zhernakova et al., 2016; Rothschild et al., 2017; David et al., 2013). Understanding the impact of diet on microbiome-mediated drug metabolism can identify dietary covariates to be corrected for in microbiome analysis and indicate the potential of tailored dietary advice during drug treatment to enhance drug efficacy. One example already in practice is the high-protein diet suggested during digoxin treatment to block inactivation of digoxin by *E*. *lenta* (Haiser et al., 2013). *A*. *muciniphila* not only enhances the response rate of PD-1/PD-L1 blockers but also exerts beneficial effect on metabolic health. High abundance of *A*. *muciniphila* is associated to low BMI, low risk of type II diabetes and a healthy lipid profile (Dao et al., 2016; Plovier et al., 2017; Everard et al., 2013). *A*. *muciniphila* has been identified as a mucin-degrading bacterium that resides in the mucus layer and that abundantly colonizes in nutrient-rich environments (Derrien et al., 2004). Inter-individual variations in *A*. *muciniphila* are mostly linked to environmental factors (Fig. 3B) (Goodrich et al., 2014). It has been reported that dietary polyphenols can promote the growth of *A*. *muciniphila* in mice (Roopchand et al., 2015) and that administration of oligofructose to genetically obese mice increased the abundance of *A*. *muciniphila* by ~100-fold (Everard et al., 2011). Moreover, metformin is found to increase SCFA-producing bacteria in the human gut, which can contribute to the therapeutic effects of metformin (Forslund et al., 2015; Wu et al., 2017). However, production of SCFAs also requires dietary fibres. Indeed, a recent dietary intervention study has revealed that weight loss in metformin users is positively associated with higher dietary fibre intake but not with total carbohydrate intake (Sylvetsky et al., 2017). Thus, certain dietary components or prebiotics can induce shifts in the gut microbiome and thereby modulate drug responses. However, we also should bear in mind that such effects can be bi-directional as drugs can also perturb the gut microbiome. In addition, the gut microbiome may also determine an individual’s response to dietary interventions (Zeevi et al., 2015).

## **SYSTEMS PROSPECTIVE IN PHARMACOGENOMICS AND PHARMACOMICROBIOMICS**

With the complex diet-drug-host-microbe interaction in mind, a great challenge lies ahead of us in predicting an individual’s response to a specific drug. This is central for successful implementation and clinical application of personalized (or precision) medicine. Two successful applications thus far are the Israeli personalized nutrition study, which predicted individual postprandial glycaemic response using a machine-learning algorithm to integrate blood parameters, dietary habits, anthropometrics, physical activity and gut microbiome (Zeevi et al., 2015), and the drug prediction algorithm, vedoNet, which incorporates microbiome and clinical data to predict the individual response to IBD treatment (Ananthakrishnan et al., 2017). To move further toward clinical applications, it is important to understand the underlying causality and mechanisms, an aim which require a systems biology approach coupling pharmacogenetics, pharmacogenomics and pharmacomicrobiomics to improve our understanding of factors that control drug pharmacokinetics at the individual level.

### **Well-characterized human cohorts**

**Figure 5:**
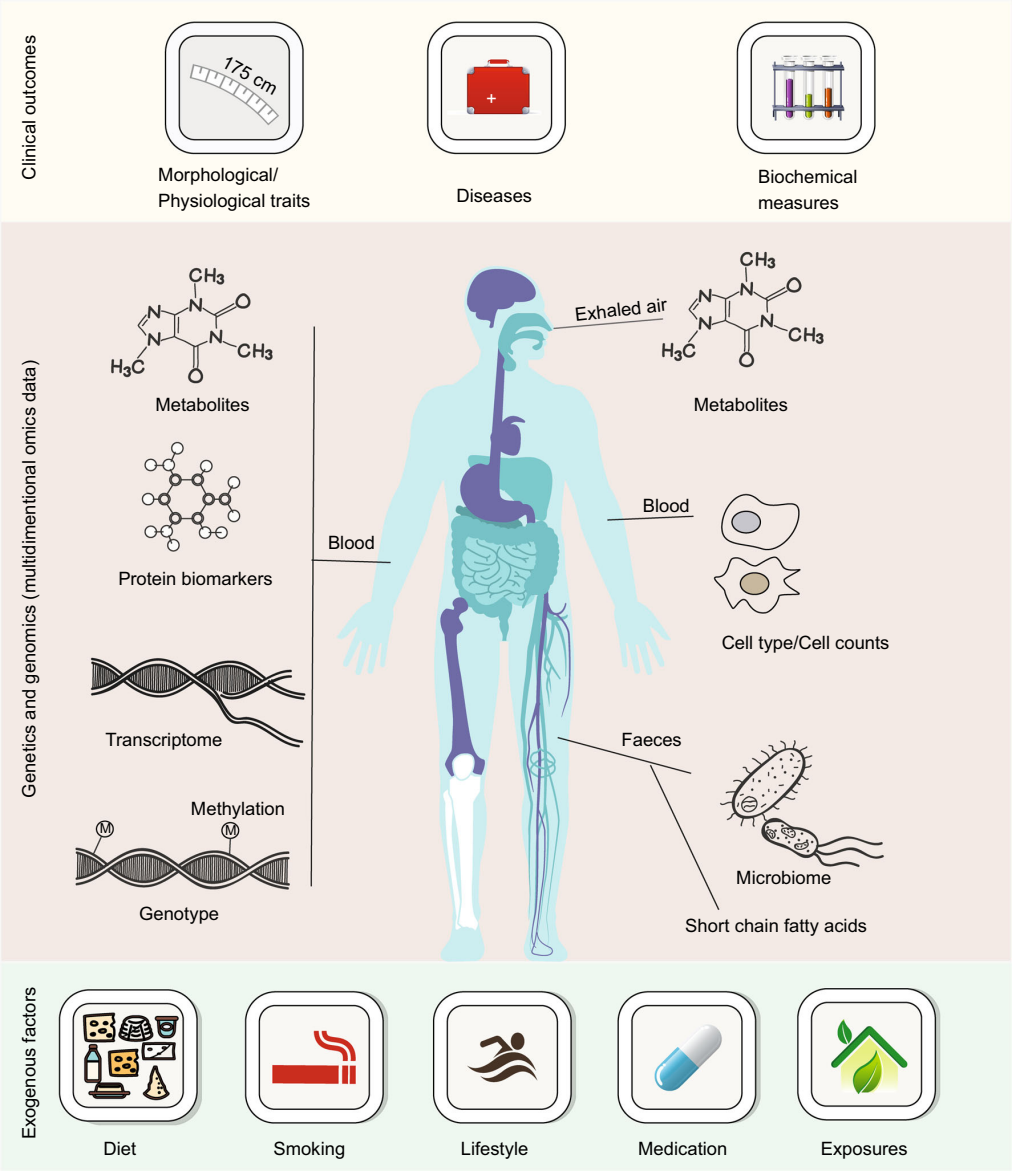
**Overview of LifeLines-DEEP cohort**. LifeLines-DEEP is a subset of 1,500 individuals from the large, prospective, population-based LifeLines cohort (*n* = 167,000 individuals). In addition to information about >2,000 exogenous factors (morphological, physiological, clinical), LifeLines-DEEP participants have been deeply profiled for multiple “omics” data layers

An “ideal” systems biology study in humans should facilitate the generation of data from the same individuals over time on multiple dimensional levels. It should include information on diet and lifestyle, living environment, presence of disease and use of drugs, and incorporate multiple omics layers via information on genetics, transcriptome, proteome, metabolome and gut microbiome. A number of large biobanks that capture gut microbiome data have now been established, including the LifeLines-DEEP cohort (Zhernakova et al., 2016; Tigchelaar et al., 2015), the UK biobank and TwinsUK cohort (Goodrich et al., 2014; Sudlow et al., 2015), the Flemish cohort (Falcony et al., 2016) and the Israeli personalized nutrition cohort (Zeevi et al., 2015). These cohorts have greatly advanced our understanding of host-microbe interactions in health and disease and of their interplay with exogenous factors. Preferably, the cohorts should have a longitudinal design in order to tackle perturbations, perform interventions and predict disease outcomes based on factors such as genetic risk, gut microbiome, molecular biomarkers, physiological traits and environmental factors. The longitudinal, prospective LifeLines cohort, for example, has been following 167,000 individuals for 10 years and will continue to do so for another 20 years. Within LifeLines, questionnaires on lifestyle, disease, drug use, quality of life, and other factors are collected regularly, and all participants undergo physical measurements every 5 years, with fasting biological samples (blood, urine, etc.) being collected at the same time. Over 2,000 phenotypic factors are recorded for each individual (Scholtens et al., 2015). In addition to this large collection of physiological and lifestyle factors, several initiatives have been set up to generate deep molecular data to enable systems biology studies (Fig. 5). For example, the LifeLines-DEEP cohort, a subset of 1,500 individuals from the LIfeLines cohort, is deeply profiled for various “omics” data on the genome, epigenome, transcriptome, proteome, metabolome and gut microbiome (Tigchelaar et al., 2015). LifeLines-DEEP serves as the foundation for systems biology and systems genetics analyses and for understanding inter-individual variation in the gut microbiome and host-microbe interaction in health and disease (Zhernakova et al., 2016; Imhann et al., 2016; Bonder et al., 2016; Fu et al., 2015; Tigchelaar et al., 2016). In recent years, two additional initiatives have been launched. One is the LifeLines DAG3 study, which is collecting oral, airway and gut microbiome data from 10,000 individuals across a wide age range (8–91 years). Metagenomic sequencing of the LifeLines DAG3 samples is underway to assess taxonomy, strain diversity and functionality. Uniquely, not only are all individuals fully genotyped but glycerol aliquots of their microbiota are also stored to enable bacterial culture for further functional studies. LifeLines DAG3 will have the power to study host-microbe interactions and will also allow studies to move from association to causality. The second initiative is the LifeLines NEXT cohort, which includes 1,500 pregnant women and their newborns and performs detailed phenotyping across the first year of life to study the development and maturation of the gut microbiome and virome and the impact of genetics and environmental factors on the developing microbial ecosystem. In all three initiatives, genetics, gut microbiome, medication use, diseases, dietary and environmental factors are available for each individual. This offers a great opportunity to systematically investigate individual variability in drug metabolism and underlying genetic, microbial, dietary factors and their interactions.

Moving from association to causality. Population-based studies with deep omics data provide powerful means for identifying risk factors in humans. Several bioinformatics-based causal inference methods, including Mendelian randomization approaches and structural equation modelling, can be used. However, this inferred causality requires further experimental validation. Transplanting the whole microbiome, specific species or a mixture into model organisms has proven to be a powerful method to illustrate causality. For instance, transferring human microbiota to germ-free mice has validated the causal role of the microbiome in mediating therapeutic effects of metformin and autoimmune therapy (Forslund et al., 2015; Wu et al., 2017; Routy et al., 2018). However, it is increasingly clear that animal models fall short in predicting pharmacokinetics in humans. It is well established that gut microbiome, metabolism and drug responses are very different between mouse and human. The knowledge generated in mouse models, even in humanized mouse models, is not directly applicable to humans. Thus several studies have conducted clinical interventions in humans to prove causality of interactions in humans, as shown by the effect of metformin (Wu et al., 2017). Yet, due to the obvious practical and ethical issues associated with human clinical studies, very little causality has actually been validated in humans. New approaches that allow for individualized drug testing *in vitro* may be set to change this landscape.

## **CULTUROMICS AND ORGAN-ON-CHIP: NEW OPPORTUNITIES FOR DEVELOPING PERSONALIZED TREATMENTS**

Individualized drug testing *in vitro* is urgently needed as a tool to aid precision medicine. Such a model should also be able to trace chemical transformation of drugs and mimic mechanical, structural, absorptive, transport and pharmaceutical properties of drugs within the gut-liver system. Given the interplay between the gut microbiome and host genome in drug metabolism, there is increasing awareness that we should take both personal microbial composition and personal genome into account when considering personalized medicine.

Microfluidic organs-on-chips are an emerging technology that mimics human organs or tissues. Differentiating human induced pluripotent stem cells (iPSCs) can give rise to different cell types that carry the genetic makeup of the iPSC-donors. An individual’s iPSCs can be programmed to become gut epithelial cells or liver hepatocytes using FGF/BMP-induced differentiation. This innovative and non-invasive technology enables functional study in the milieu of an individual’s genetic background, which holds great promise in disease-modelling and drug-testing applications (Bhatia and Ingber, 2014; Huh et al., 2011). The potential of this approach has been shown in various applications using organoid models and organ-on-chips (Trietsch et al., 2017; Kim et al., 2012; Takayama et al., 2012; Takebe et al., 2014). For instance, a liver-on-a-chip has been engineered that mimics heterotypic interaction by separating iPSC-derived hepatocytes from the active flow microchannels designed to resemble the natural endothelial barrier of the liver sinusoid (Huh et al., 2011). The device has now been proven to maintain metabolic activity of the hepatocytes for over 7 days and to permit metabolic analysis.

Most gut microbes are strictly anaerobic and, for much of the last century, fewer than 30% of them could be cultured in the laboratory, which made functional studies impossible. With advances in culture-independent next-generation sequencing technologies, we have started to gain more insights into the composition and function of gut microbes based on their DNA sequencing. These bioinformatics-based approaches have yielded many insights into the underlying mechanisms. However, to further validate these mechanisms requires functional studies using a culture-based approach. In recent years bacterial culture technologies have been developed that now allow around 80% of gut microbes to be cultured (Lagier et al., 2016), making functional validation of gut microbe processes finally possible. For instance, bacterial metabolism of corticosteroids and ranitidine has been determined using *in vitro* cultures (Yadav et al., 2013; Basit and Lacey, 2001). These cultures attempt to closely mimic the colonic environment for specific strains of bacteria and host-microbe interactions. Drugs are then added to assess their impact on bacterial growth and metabolism and, *vice versa*, how bacteria chemically transform these drugs.

**Figure 6:**
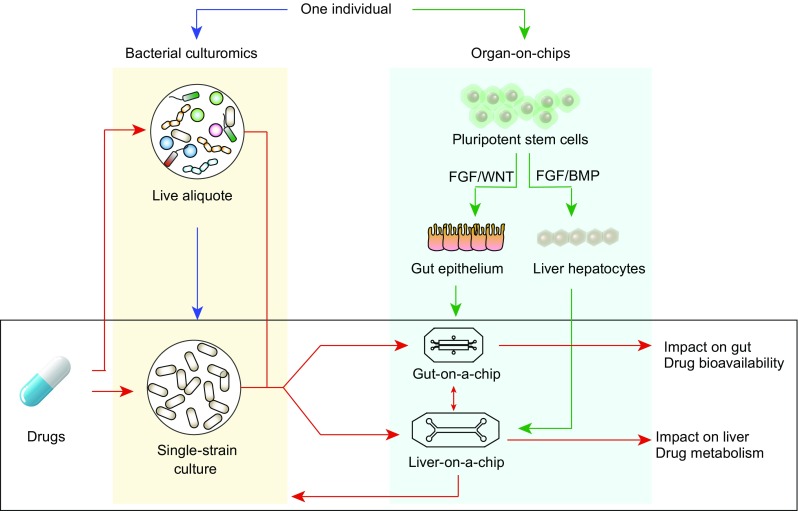
**Individual-based drug testing**. Advance of bacterial “culturomics”, development of organs-on-chip and high-throughput metabolism and pharmacokinetic analyses, will enable individual-based *in vitro* drug testing in the near future. For this purpose, liver gut microbiome can be collected for culturing. This can be done either on whole community level or on individual species or strain level (blue arrows). Currently, organs-on-chips are emerging as a next-generation drug-testing model system. Non-invasive collection of urine leads to human induced pluripotent stem cells from which we can generate different types of tissue cells (e.g., gut epithelial cells or hepatocytes) (green arrows). These cells will have exactly the same genetic background. Coupling cultured bacteria and organs-on-chip offers a high potential to conduct individual-based drug testing, by taking into consideration both an individual’s own genome and his/her metagenome (red arrows)

With the advance of organs-on-chips and the bacterial culturomics, we anticipate that *in vitro* models in the next-phase of personalized medicine will also be able to couple personalized genome and metagenome and to conduct individual-based drug testing on cultured bacteria, gut epithelial cells and hepatocytes simultaneously (Fig. 6). We will thus be able to apply drug breakdown products of bacterial enzymes or other bacterial metabolites to the gut-on-a-chip and liver-on-a-chip to further investigate their effect on the host cells. Moreover, drug metabolites produced by hepatic enzymes can also be applied to the gut microbiome and gut epithelial cells to evaluate whether their enzyme can re-activate drugs and cause adverse events.

## **CONCLUSIONS**

The microbes residing in the human gut encode a broad diversity of enzymes, greatly expanding the repertoire and capacity of metabolic reactions in the human body that can be involved in xenobiotic metabolism, including that of dietary components and drugs. The gut microbiome is thus emerging as an important player in personalized medicine. Several papers have discussed the role of the gut microbiome on drug efficacy and toxicity (Alexander et al., 2017). Here, we are considering the host-microbe-drug interactions and the impact of dietary factors. We further propose a comprehensive analysis framework that combines well-characterized human cohorts and innovative *in vitro* model systems to study this complex interaction. Notably, in contrast to human genetic make-up, gut microbiota can be modulated. Pharmacomicrobiomics thus may have at least two global clinical applications: 1) to combine personal microbiome and genetic profiles to better predict an individual’s medication response; and 2) to modulate the gut microbiome to improve drug efficacy on the individual level. However, given the great diversity of microbial composition, its broad function in the host and the complex drug-diet-microbe-host interactions, a systems-based approach and individualized drug testing systems are needed to further understand the underlying causalities and mechanisms. The recent advance in cutting-edge, state-of-art technologies in bacterial culturomics and individualized organs-on-chips, together with exponential growth of databanks and biobanks holding vast amounts of information about the same individual, will enable the development of the next phase in personalized medicine.
